# A systematic investigation of four ports MIMO antenna depending on flexible material for UWB networks

**DOI:** 10.1038/s41598-022-18551-8

**Published:** 2022-08-23

**Authors:** Ahmed A. Ibrahim, Mohamed I. Ahmed, Mai. F. Ahmed

**Affiliations:** 1 Electronics and Communications Engineering Department, Minia University, El-Minia, Egypt; 2 Microstrip Department, Electronics Research Institute, Giza, Egypt; 3 Department of Electronics and Comm. Engineering, Faculty of Engineering, Zagazig University, Zagazig, Egypt

**Keywords:** Engineering, Electrical and electronic engineering

## Abstract

A flexible quad-port MIMO antenna with good isolation features with both flat and bending configurations is presented and investigated in this work. The single unit of the MIMO is composed of a crescent-shaped monopole antenna connected with a curved coplanar waveguide (CPW) fed to enhance the operating bandwidth. A thin and flexible Roger 3003 material with thickness = 0.13 mm, εr = 3, and tan δ = 0.001 is used. To improve the isolation between ports which in turn improves the performance of the MIMO system, the single unit antenna is repeated four times and placed orthogonal to each other. A 54 mm × 54 mm × 0.13 mm (0.63λo × 0.63λo × 0.0015λo @ 3.5 GHz) is the total size of the quad ports MIMO antenna. The flexible MIMO antennas in both flat and bending layouts are simulated, tested and the outcomes achieved S_11_ < − 10 dB from 3.5 GHz up to 11 GHz with mutual coupling ≤ − 17 dB between ports. The radiation patterns of the MIMO antenna are tested with 5 dB peak gain and with semi-omnidirectional and bidirectional patterns in both two planes. The Diversity Gain (DG) values ≥ 9.9 dB through the designed working band, Envelop Correlation Coefficient (ECC) lower than 0.03 from 3.5 GHz to 4 GHz and lower than 0.01 from 4 to 11 GHz, and Channel Capacity Loss (CCL) value ≤ 0.5 bit/s/Hz over the worked band are calculated and extracted in flat and bending configurations and achieved suitable values which support the suggested antenna in the UWB flexible networks.

## Introduction

Over the last few years, technology has rapidly changed, necessitating a considerable increase in data rate and development in the wireless networks industry, both of which have become critical in meeting the massive demand for data rate^1^. Therefore, the unlicensed band from 3.1 to 10.6 GHz is approved by the Federal Communications Commission (FCC)^2^. In the UWB systems, the antenna has the main part for communication. So, the antenna should have a compact size, low price, covered the operating bands, and has distinctive radiation behavior^3–6^. However, it is communicated through short distances and the multipath problem is one of the UWB technology problems. So, a Multiple-input multiple-output (MIMO) antenna is introduced. The quality of signal and high capacity without increasing the transmitted power is considered the main contribution of the MIMO system. By introducing and integrating several antennas in the transmitting and receiving ends of the UWB systems, multipath fading reduction and channel capacity improvement can be achieved^7,8^. Hence the antenna in the MIMO system must be small-sized and portable, in addition to being isolated and uncorrelated to one another^9–11^. To decrease the overall size of the MIMO system, the proposed antennas should be added with a small distance between them. However, adding an antenna with a small distance increase the mutual coupling between elements and decrease the isolation between ports. as well, the efficiency of the individual antenna can be affected^12^ which leads to the use of decoupling structures to reduce the mutual coupling which in turn increases the complexity of the design^10,13^. Both 2-port and 4-port with rigid and flexible substrates and with suitable isolation can be utilized in the MIMO systems^13–23^.

In^13^, 4 ports monopole antennas with four stubs between elements placed on a rigid FR-4 substrate operating from 3.2 to 12 GHz and producing isolation and peak gain higher than 17 dB and 4 dBi. A four ports MIMO antenna printed on a rigid FR-4 substrate with adding the elements orthogonally to each other is designed to operate from 3.03 to 10.74 GHz and achieve isolation higher than 20 dB and a peak gain of 5 dBi is investigated in^14^. As well, a small size 4 ports monopole nonflexible antenna with adding the elements orthogonally to each other to reduce the isolation is introduced in^15,16^. In^17,18^, a 2 ports MIMO antenna printed on a rigid FR-4 substrate with a large size operating at the UWB frequency band is discussed and investigated. A dual wideband 4 ports MIMO antenna operated from 1.5 to 3.8 GHz and from 4.1 to 6.1 GHz placed on jeans flexible substrate and produced high isolation by presenting meandered line decoupling structure between antenna elements is discussed in^19^. In^20^, a 4 ports monopole antenna was placed on a flexible Rogers's substrate operating from 3.2 to 14 GHz and producing isolation and peak gain higher than 22 dB and 4.5 dBi. In^21,22^, and^23^ a 2 ports MIMO antenna added on a flexible substrate with a large size operating are presented and investigated.

A quad-port MIMO antenna added on a flexible Rogers's 3003 substrate with thickness = 0.13 mm, εr = 3, and tan δ = 0.001 is used. It covered the UWB frequency band suggested in this paper. The antenna has good isolation features in both flat and bending configurations. The single unit of the MIMO is composed of a crescent-shaped monopole antenna connected with a curved coplanar waveguide (CPW) fed to enhance the operating bandwidth. The isolation between units is enhanced by placing the four units orthogonally to each other without utilizing any decoupling structure to simplify the suggested design. The MIMO parameters are calculated and extracted in flat and bending configurations and achieved suitable values. Finally, the reported work advantages and novelty can be summed up in the next points.

- The suggested structure is flexible with a thin substrate and achieved the isolation of more than 17 dB between ports without decoupling structure and with wideband and reasonable gain which support the suggested antenna in the UWB flexible networks.
- The antenna is flexible and compact which can offer easy integration with other components and save more space.
- The antenna operates in a good way under the bending conditions in the term of S-parameters ECC, DG, and CCL which confirms the stability of the suggested antenna under bending conditions.
- A flexible MIMO antenna in comparison with other flexible and rigid designs to confirm the performance of the suggested MIMO antenna shows that the suggested antenna has a miniaturized size with a thin substrate than the flexible and rigid substrates in^13–23^ with a suitable level of isolation, gain, and ECC values that support it in the UWB flexible networks applications.

## Flexible single unit antenna design procedures

**Figure 1:**
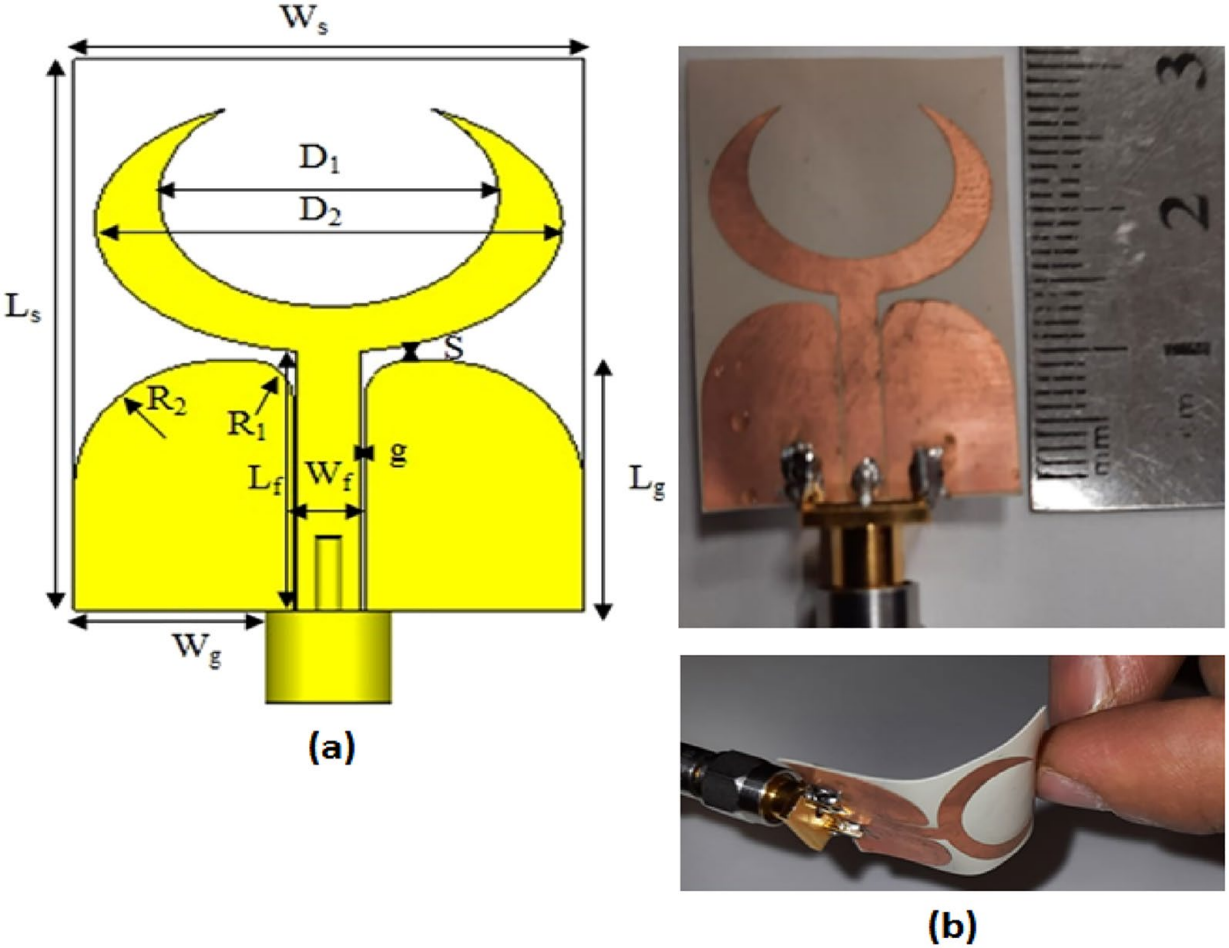
Flexible single antenna configuration, (**a**) 2 D view, (**b**) fabricated photo prototype.

Figure 1a illustrates the single unit configuration of the suggested UWB MIMO antenna whilst the fabricated photo prototype is shown in Fig. 1b. The antenna is composed of a crescent-shaped monopole antenna which consists of a difference between the two ellipses connected with a curved 50 Ω CPW fed by adjusting the gap (g) between the feed line and the curved ground as well as the feed line width (Wf) to enhance the operating bandwidth. The crescent-shaped and the ground plane are added on the same side of a thin and flexible Roger 3003 material with thickness = 0.13 mm, εr = 3, and tan δ = 0.001 to achieve the overall size of 24 mm × 30 mm (0.28λo × 0.35λo @ 3.5 GHz). The antenna is designed and optimized using the Microwave Studio CST program and its optimized dimensions are tabulated in Table 1.

**Table 1:** The dimensions of the single unit UWB antenna.

Ws	Ls	D1	D2	W_f_	L_f_	Wg	Lg	R_1_	R_2_	S	g
24	30	16	22	3.5	14	10	13	1.8	6.7	0.6	0.2

**Figure 2:**
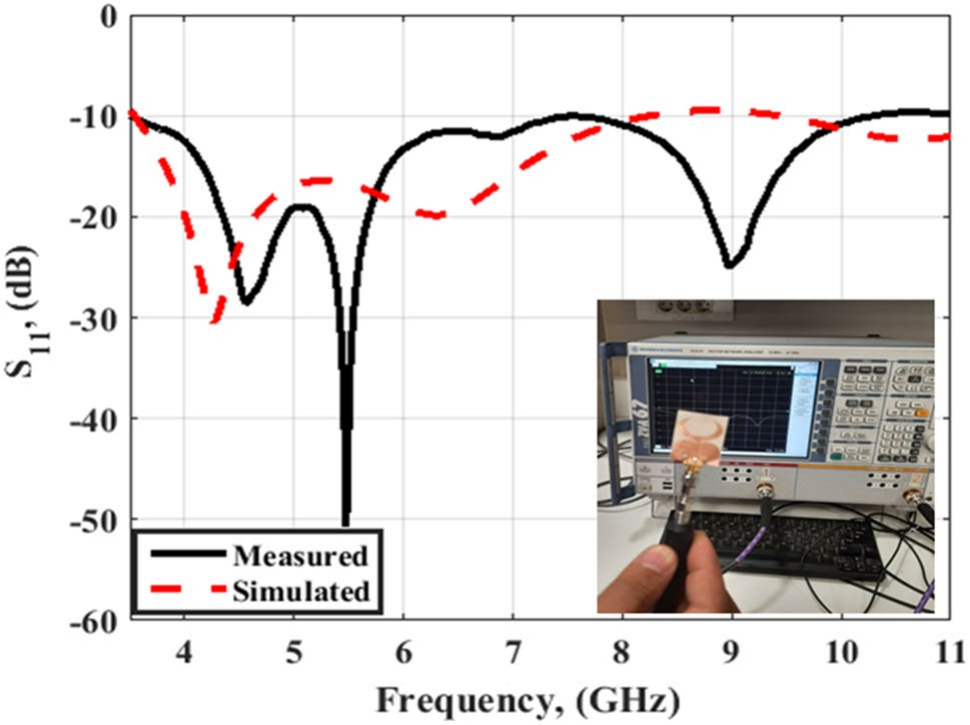
S_11_ outcomes of the flexible single antenna.

The Rohde & Schwarz ZVA 67 vector network analyzer (VNA) is utilized in the testing process and the tested and simulated outcomes are shown in Fig. 2. The antenna has a frequency band beginning from 3.5 GHz up to 11 GHz with S_11_ < − 10 dB with a reasonable trend between the two outcomes. Also, there is a shift between the two outcomes; this is because of the fabrication setup, the human error during the measurements because of the small and thin antenna configuration as compared to the heavy SMA connector which needs more accuracy during the measurement setup.

**Figure 3:**
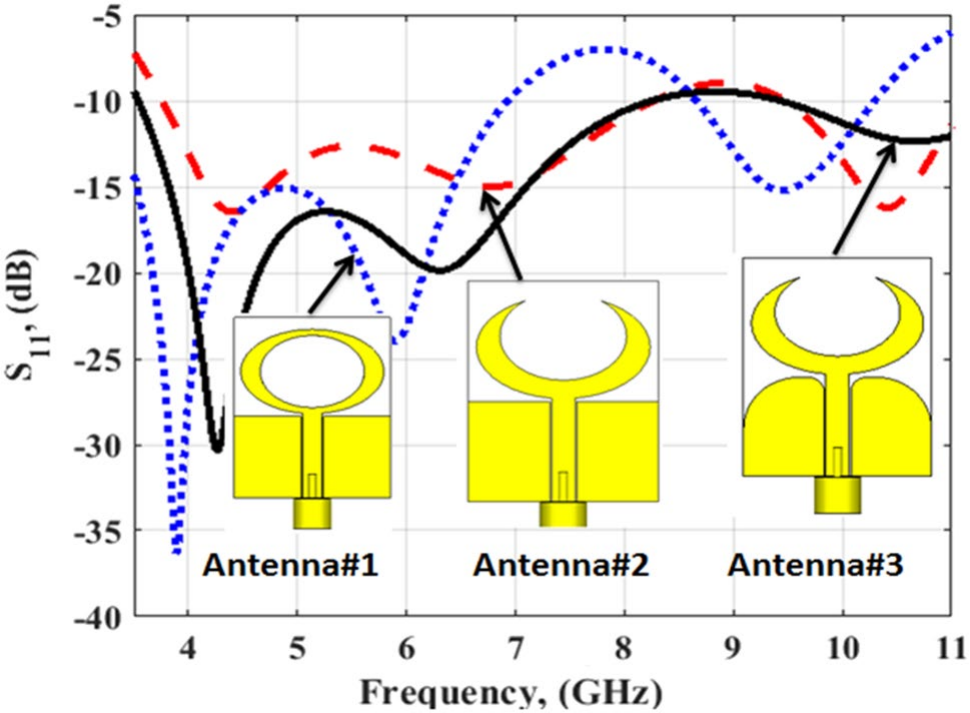
Flexible single antenna developments.

**Figure 4:**
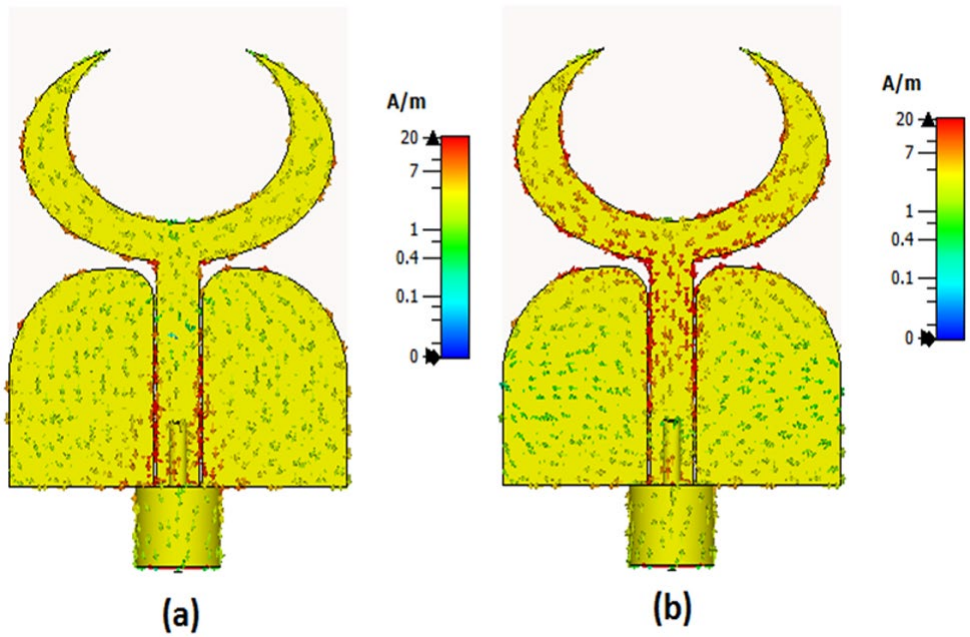
The current distribution outcomes (**a**) @ 4.5 GHz, (**b**) @8.5 GHz.

**Figure 5:**
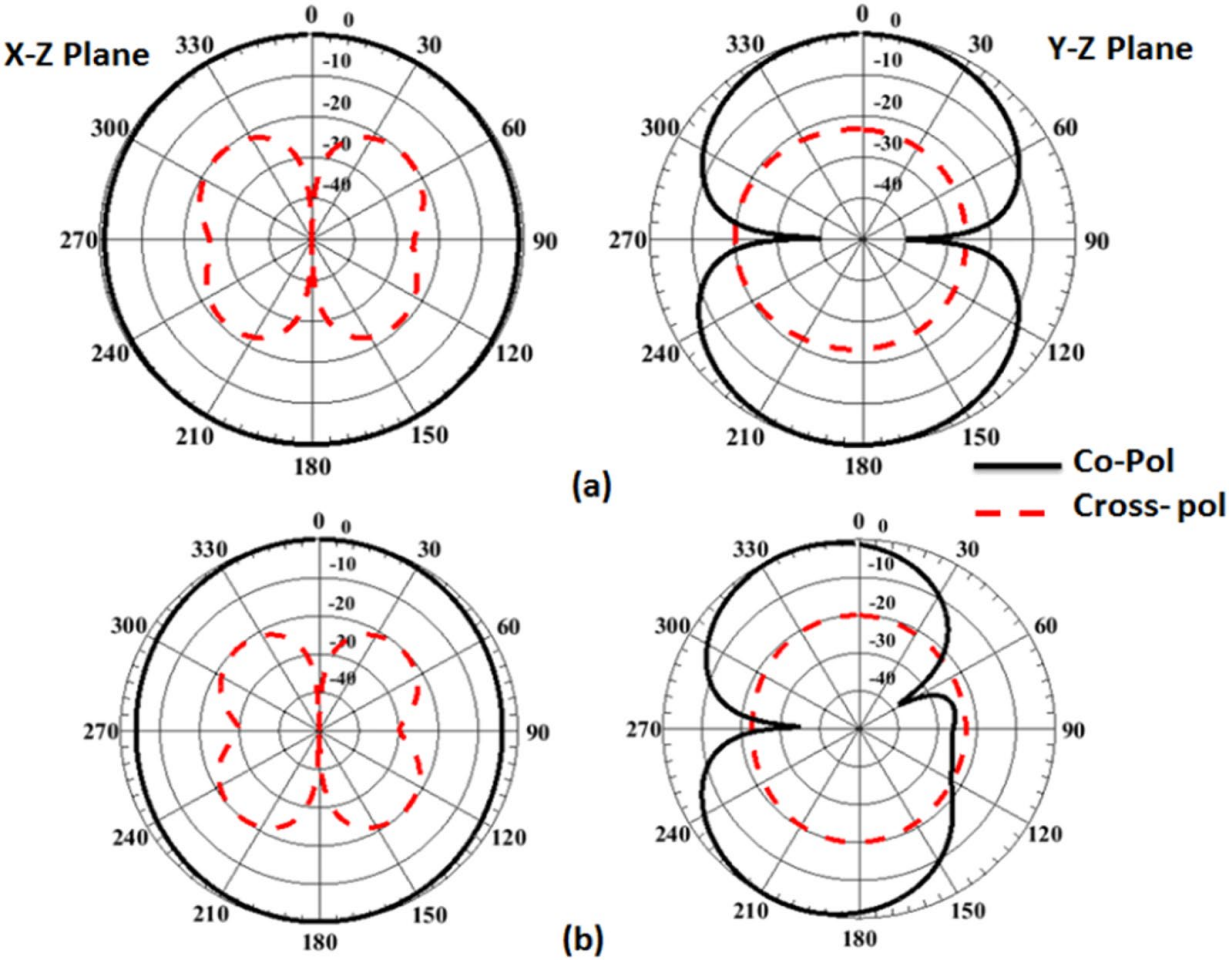
The Co/Cross- pol of the flexible single antenna (**a**) @ 4.5 GHz, (**b**) @8.5 GHz.

**Figure 6:**
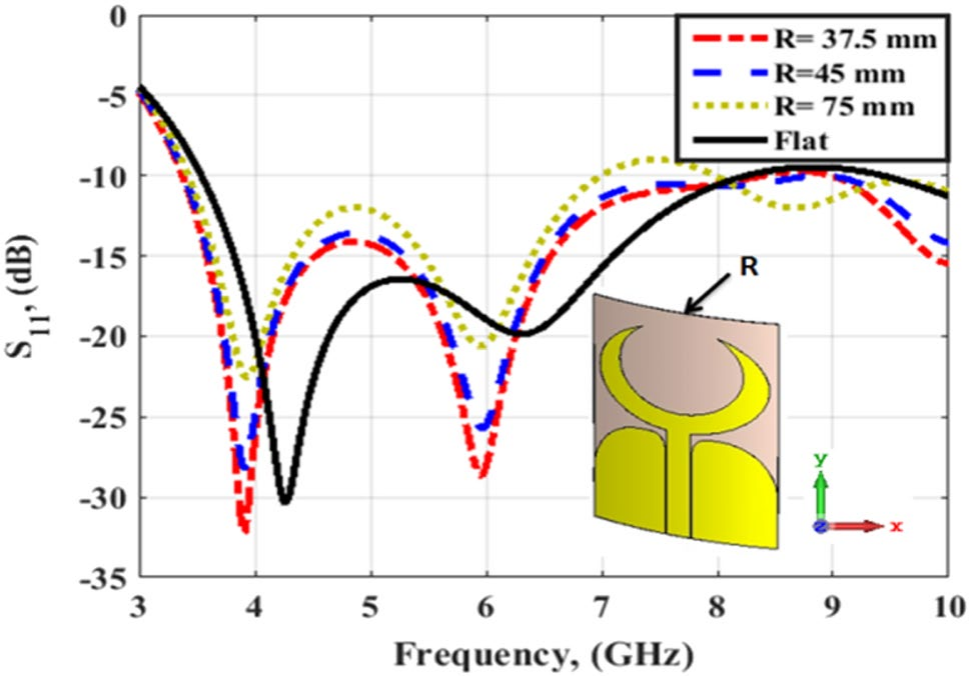
The flexible single antenna bending effect with three different radiuses.

Figure 3 illustrates the development of the single unit antenna starting from the initial design to the proposed structure. Antenna #1 (two elliptical-shaped with different radius with the straight ground). The two elliptical-shaped are subtracted to achieve the desired lower frequency band. The simulated S_11_ (the blue dotted line) shows that the antenna is operated at two bands from 3 to 6.5 GHz and from 8.5 to 10.5 GHz. Then the crescent radiator shape is generated by increasing the radius of the inner ellipse and with the same straight ground (antenna#2). The simulated S_11_ (the red dashed line) shows that the antenna is operated from 3.8 to 11 GHz. Finally, by using the curved ground (antenna#3), the antenna matching is improved especially at the lower frequency band and the S_11_ (the black solid line) achieves a frequency band from 3.5 to 11 GHz. Further, the current distribution for the single unit antenna at two frequencies 4.5 and 8.5 GHz are shown in Fig. 4. From these results, the current distributions are collected around the crescent radiator which confirms its responsibility for the radiation. Figure 5 illustrates the Co polarization/Cross polarization of a single antenna at 4.5 and 8.5 GHz and the two x–z and y–z planes. It is seen that around a 20 dB difference between the co and the cross-polarization in both planes. The single unit antenna performance under bending conditions with different radiuses around the y- axis is illustrated in Fig. 6. The antenna has simulated outcomes with S_11_ ≤ − 10 dB from 3.3 GHz up to 11 GHz at different values of R. When R has increased the matching is slightly affected as the green dotted curve. Also, it can be said that the bending process around the y- axis has a small effect on the frequency response.

## Suggested flexible four ports configuration

### Flat configuration

**Figure 7:**
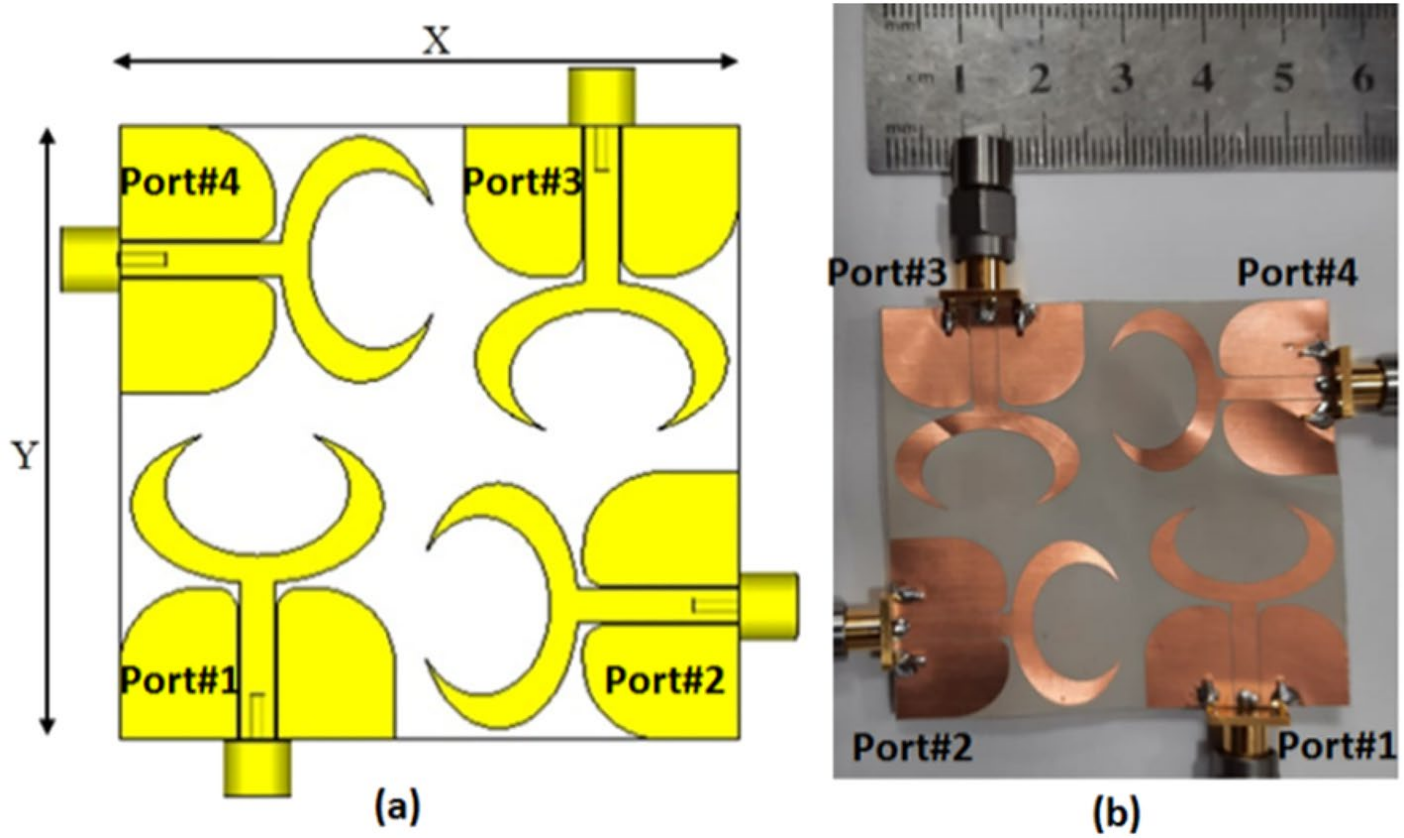
Flexible MIMO antenna configuration (**a**) 2 D view, (**b**) fabricated photo prototype.

**Figure 8:**
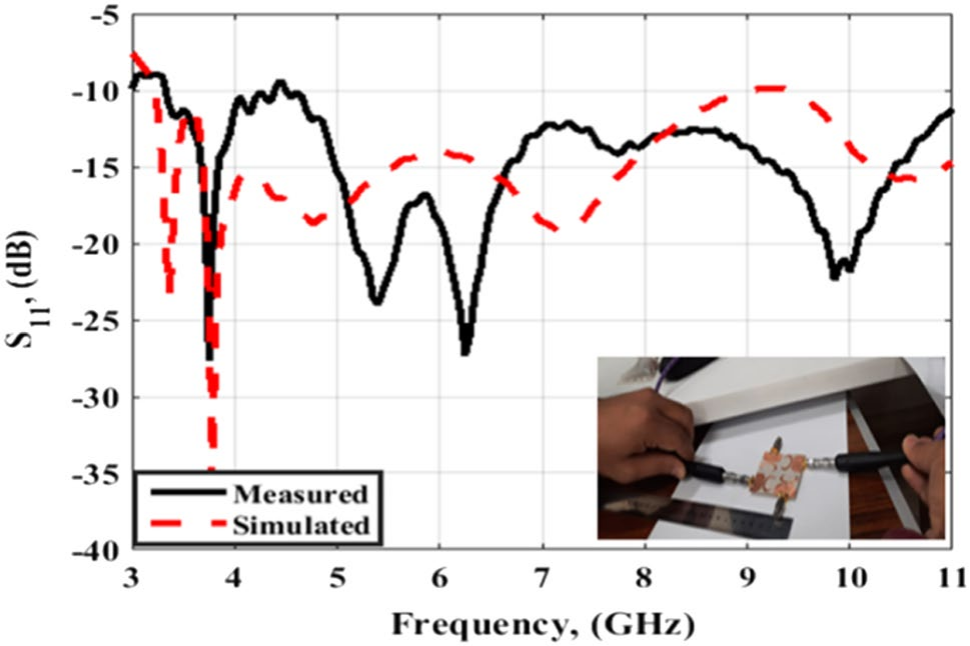
S_11_ outcomes of the flexible MIMO antenna.

**Figure 9:**
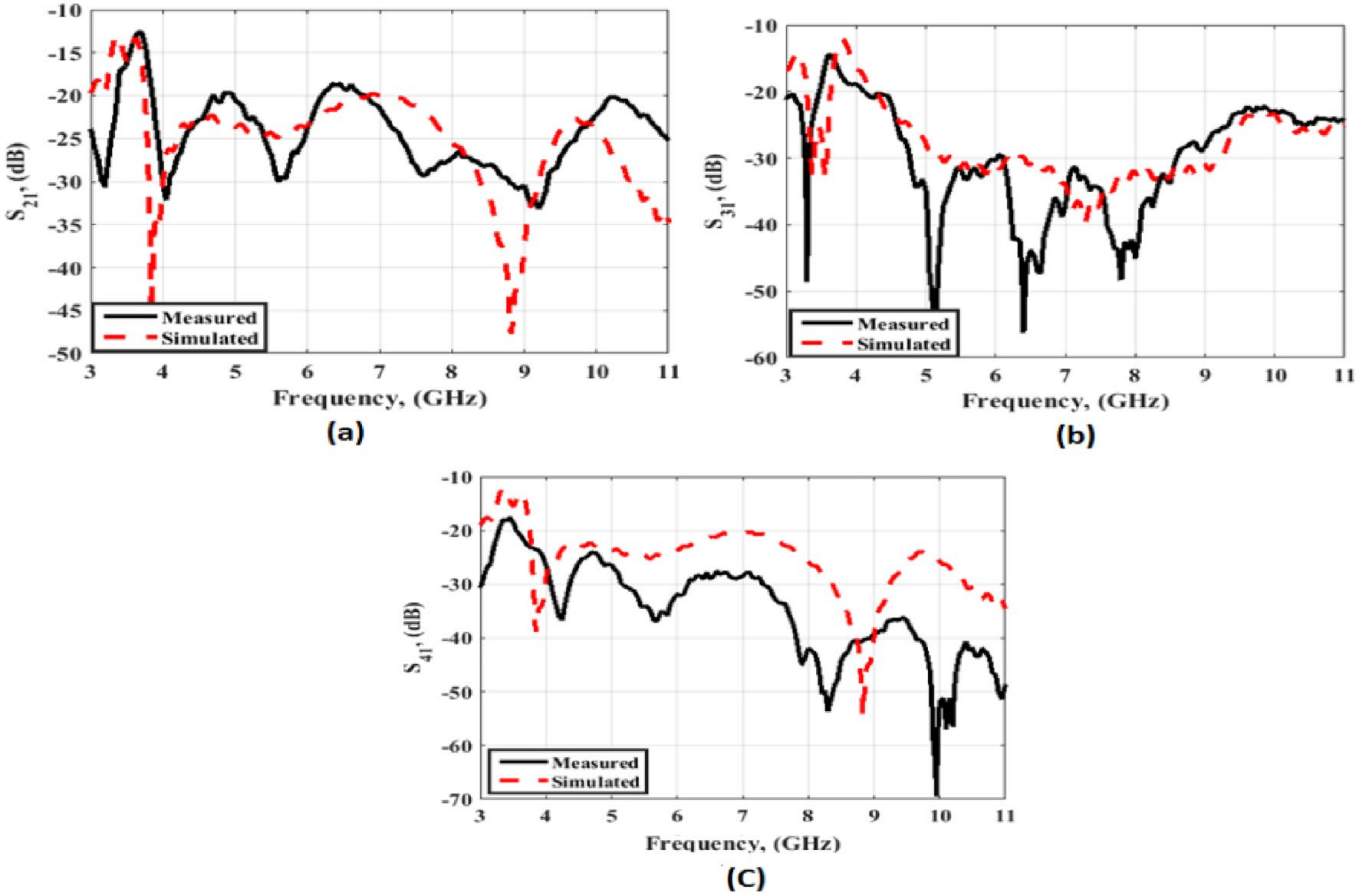
Transmission coefficients outcomes of the flexible MIMO antenna (**a**) S_21_, (**b**) S_31_, (**c**) S_41_.

The suggested crescent-shaped monopole antenna is repeated four times and placed orthogonal to each other to improve the isolation between ports which in turn improves the performance of the MIMO system as illustrated in Fig. 7. The four antenna units are added close to each other to reduce the overall antenna size with a 3.7 mm (0.04λo @ 3.5 GHz) separation between the antenna radiator's edges. The suggested flexible quad ports MIMO antenna has a total size of X = Y = 54 mm × 54 mm (0.63λo × 0.63λo @ 3.5 GHz). As well, the fabricated prototype photo is shown in Fig. 7b. Because of the symmetry of the configuration only port 1 is displayed. The flexible MIMO is tested using Rohde & Schwarz ZVA 67 VNA at port 1 and the tested outcomes (S_11_, S_21_, S_31_ and S_41_) are shown in Figs. 8 and 9. The antenna has simulated and tested S_11_ outcomes < − 10 dB from 3.5 to 11 GHz with a reasonable tendency and slight shift between them. This is because of the stated reasons which are written in the previous section. Figure 9 illustrates the simulated and tested outcomes of the transmission coefficients of the flexible MIMO antenna at port 1. The antenna introduces an isolation level lower than 20 dB from 4 GHz up to 11 GHz while it has lower than 15 dB from 3.5 to 4 GHz. Also, reasonable matching between the simulated and testes outcomes is observed.

**Figure 10:**
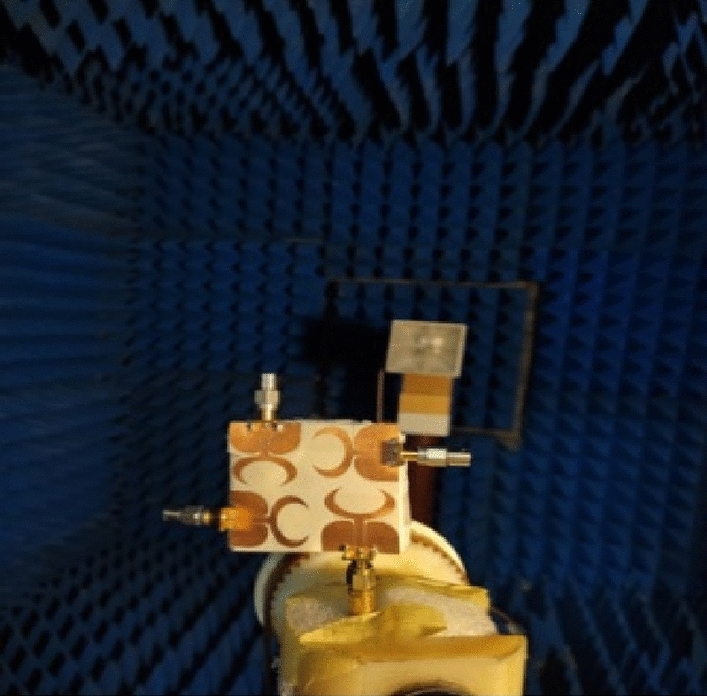
Radiation patterns testing setup of the flexible MIMO in flat configuration inside the anechoic chamber.

**Figure 11:**
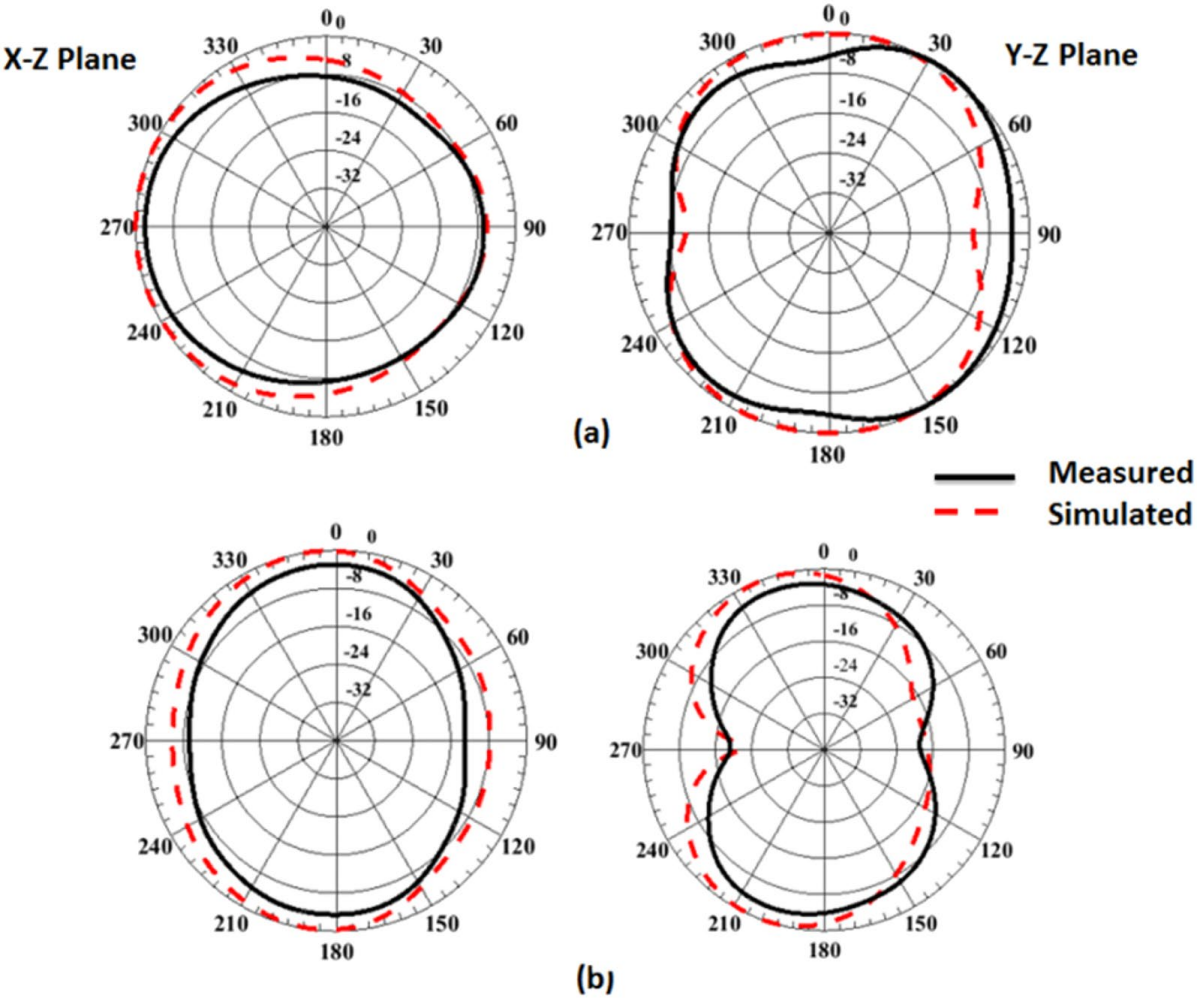
Port #1 radiation patterns simulated and testing outcomes of the flexible MIMO antenna in flat configuration (**a**) @4.5 GHz, (**b**) @ 8.5 GHz.

The suggested antenna is tested at port 1 whilst the matched 50 Ω loads are presented for the other ports to produce the radiation pattern and gain features inside an anechoic chamber and the testing setup is illustrated in Fig. 10. The normalized radiation patterns of the antenna have been tested at 4.5 GHz and 8.5 GHz in the x–z and y–z planes. The semi-omnidirectional patterns are introduced at the x–z plane while the bidirectional patterns are presented in the y–z plane with a reasonable matching between them as illustrated in Fig. 11.

**Figure 12:**
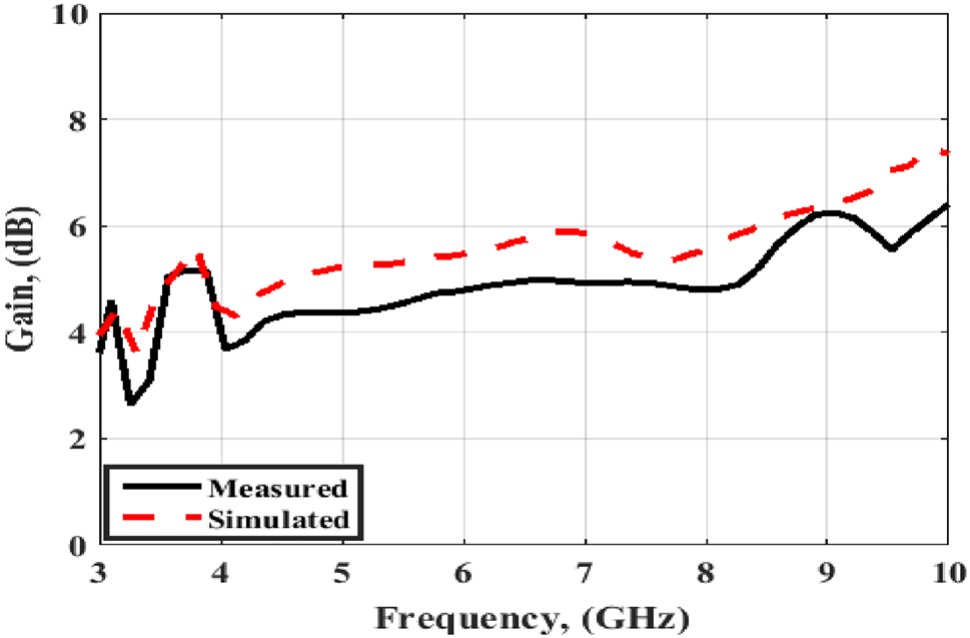
Port #1 peak gain simulated and testing outcomes of the flexible MIMO antenna in a flat configuration.

Finally, the simulated and tested results of the peak gain are displayed in Fig. 12. The antenna has tested gain ranging from 4 dBi at the beginning to 6 dBi at the end of the band with a good matching between the results.

### Bending configuration

**Figure 13:**
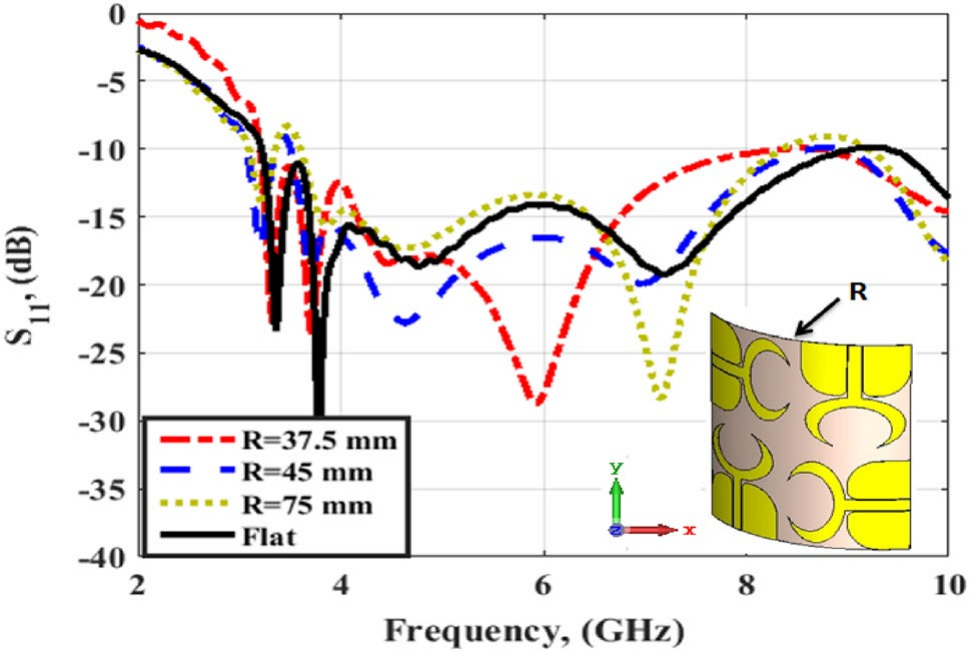
The flexible MIMO antenna bending effect with three different radiuses.

To show the effect of the bending effect on the flexible MIMO antenna performance, the previous flat MIMO antenna is subjected to bending with three different radiuses around the y-axis as shown in Fig. 13, and its S_11_ performance is displayed in the same figure. The antenna has simulated outcomes with S_11_ ≤ − 10 dB from 3 GHz up to 11 GHz at different values of R. When R has increased the matching is slightly affected especially at the beginning of the band (the green dotted curve). Also, it can be said that the bending process around the y- axis has a small effect on the frequency response.

**Figure 14:**
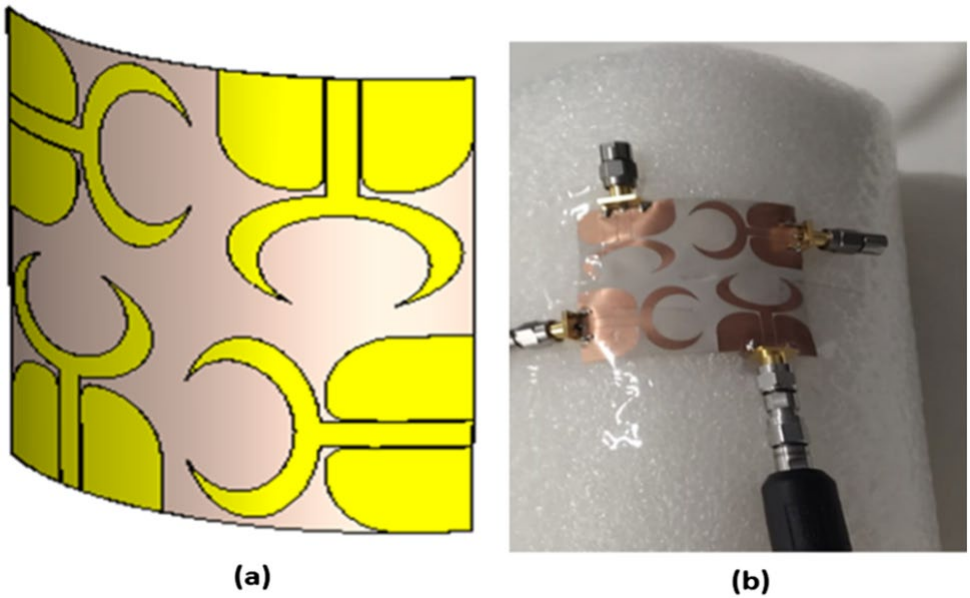
Flexible MIMO antenna configuration with radius R of 45 mm (**a**) 2 D view, (**b**) fabricated photo prototype.

**Figure 15:**
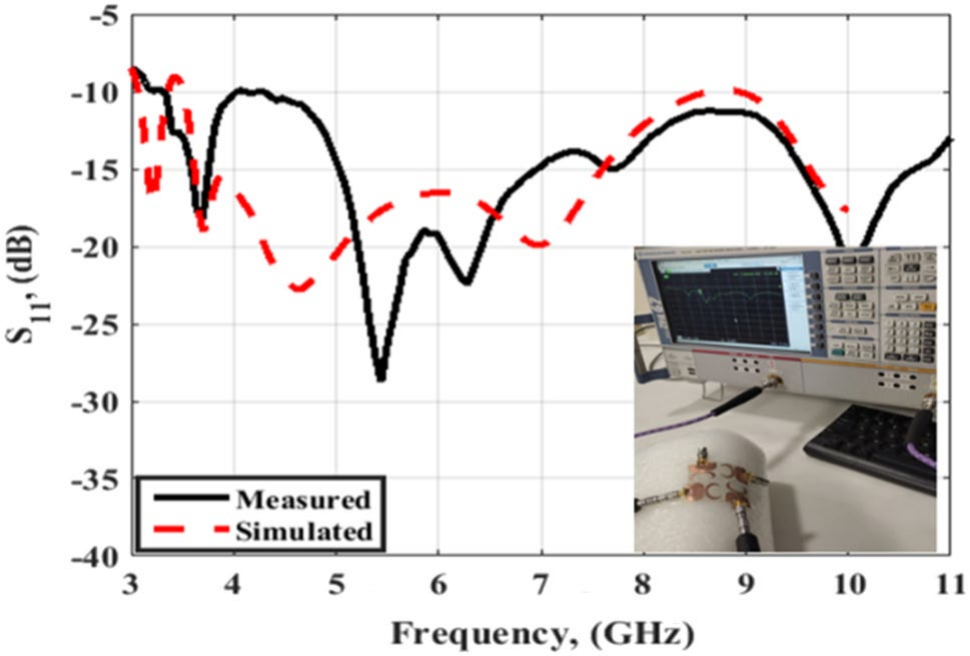
S_11_ outcomes of the flexible MIMO antenna in bending configuration with R = 45 mm.

**Figure 16:**
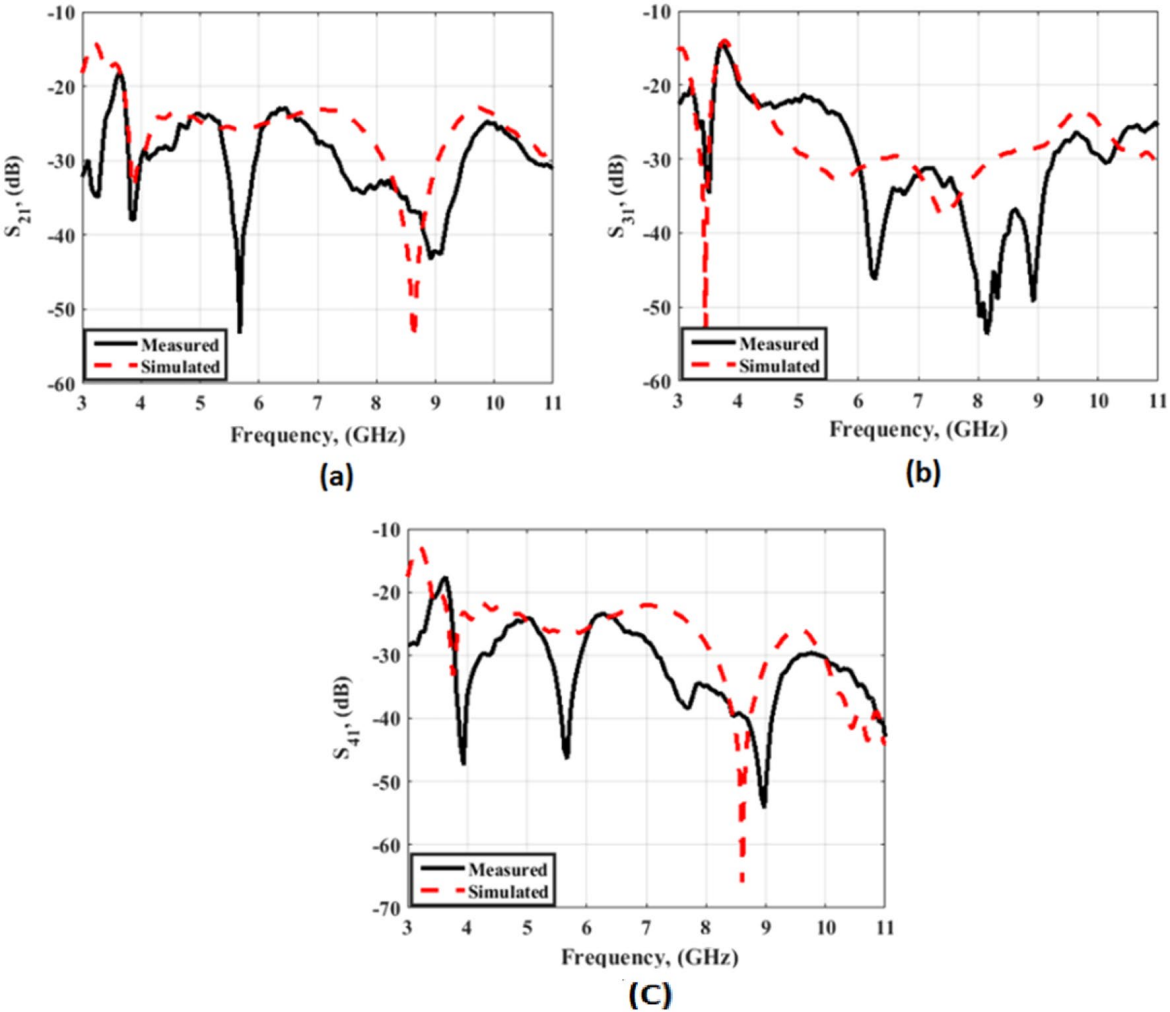
Transmission coefficients outcomes of the flexible MIMO antenna in bending configuration with R = 45 mm (**a**) S_21_, (**b**) S_31_ and (**c**) S_41_.

A radius R of 45 mm is selected for the testing as shown in Fig. 14. A cylindrical foam layer of polystyrene with εr = 1.03 is utilized to rotate the flexible MIMO around it as illustrated in Fig. 14b. The previous ZVA 67 VNA as illustrated in Fig. 14b is utilized to test the MIMO antenna at port 1 and the tested outcomes are displayed in Figs. 15 and 16. The antenna has tested outcomes beginning from 3.3 GHz up to 11 GHz with S_11_ < − 10 dB and its result has a reasonable matching to the simulated one. The S_11_, S_21_, S_31_ and S_41_ outcomes introduced at port 1 are observed in Fig. 16. The MIMO antenna achieved an isolation level lower than 20 dB from 4 GHz up to 11 GHz while it has lower than 15 dB from 3.3 to 4 GHz. Also, a reasonable tendency between the simulated and testes outcomes is accomplished.

**Figure 17:**
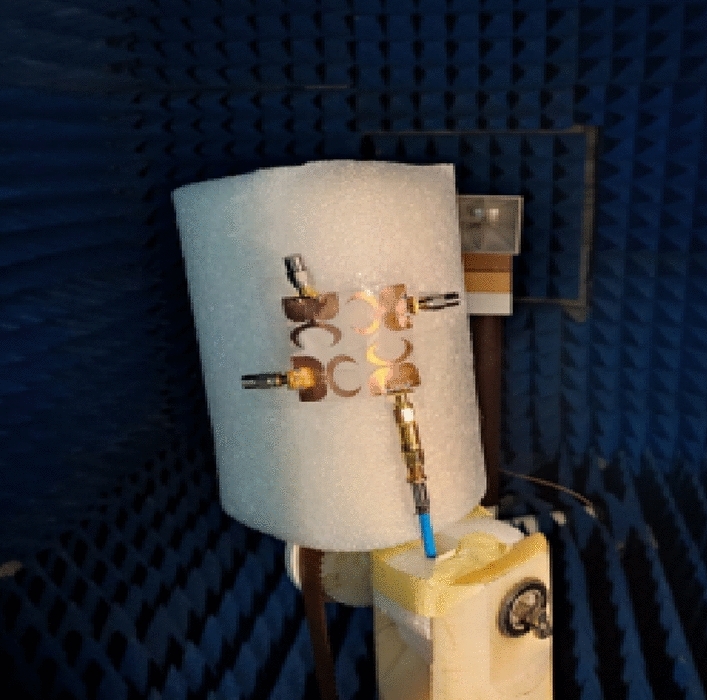
Radiation patterns testing setup of the flexible MIMO antenna in bending configuration with R = 45 mm inside the anechoic chamber.

**Figure 18:**
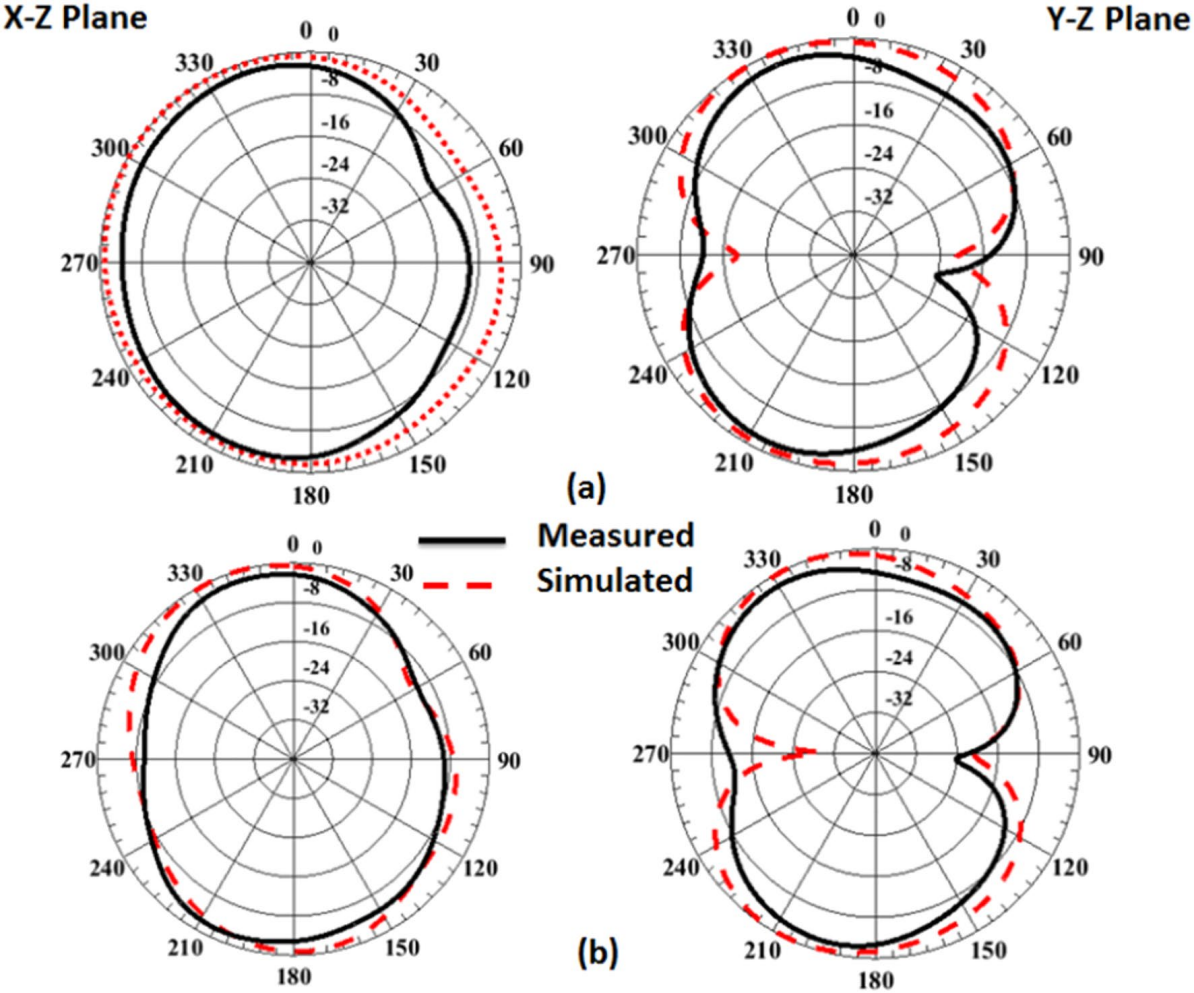
Port #1 radiation patterns simulated and testing outcomes of the flexible MIMO antenna in bending configuration with R = 45 mm (**a**) @4.5 GHz, (**b**) @ 8.5 GHz.

**Figure 19:**
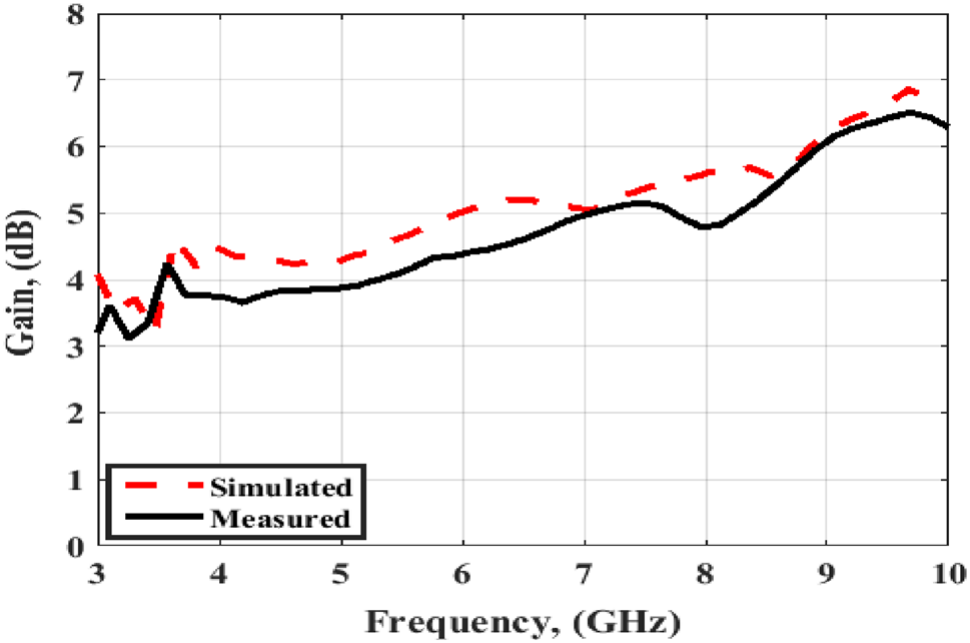
Port #1 peak gain simulated and testing outcomes of the flexible MIMO antenna in bending configuration with R = 45 mm.

Also, as the flat configuration discussed before, the curved antenna is tested to produce the radiation pattern and gain features inside an anechoic chamber and the testing setup is illustrated in Fig. 17. The normalized radiation patterns of the antenna have been tested at 4.5 GHz and 8.5 GHz in the x–z and y–z planes. The semi-omnidirectional patterns are introduced at the x–z plane and the bidirectional patterns are presented in the y–z plane with a reasonable matching between them. While the pattern is slightly tilted in the y–z plane, this is because of the bending effect around the cylindrical foam layer as shown in Fig. 18. The simulated and tested results of the peak gain are shown in Fig. 19. The antenna has tested gain ranging from 3 dBi at the beginning to 6.3 dBi at the end of the band with a good tendency between the results at R = 45 mm.

## MIMO performance investigations

The performance of the MIMO system and its diversity feature is evaluated by calculating and extracting the ECC, DG, and CCL main parameters of the flexible MIMO antenna. The isolation between ports can be checked by the ECC parameter. Lower values of the ECC reflect the high MIMO realization. The ECC values lower than 0.5 can be accepted. As well, the ECC values can be taken and calculated from the S-parameters and far-fields outcomes as (1) and (2)^24,25^.

1 $$ECC = \rho_{e} = \left| {\rho_{ij} } \right| = \frac{{\left| {{\text{S}}_{{{\text{ii}}}}^{ * } {\text{S}}_{{{\text{ij}}}} + {\text{S}}_{{{\text{ji}}}}^{ * } {\text{S}}_{{{\text{jj}}}} } \right|^{{2}} }}{{\left( {{1} - \left( {\left| {{\text{S}}_{{{\text{ii}}}} } \right|^{{2}} + \left| {{\text{S}}_{{{\text{ji}}}} } \right|^{{2}} } \right)} \right)\mathop {}\limits^{{}} \left( {{1} - \left( {\left| {{\text{S}}_{{{\text{jj}}}} } \right|^{{2}} + \left| {{\text{S}}_{{{\text{ij}}}} } \right|^{{2}} } \right)} \right)}}$$

2 $$ECC = \rho_{e} = \frac{{\left| {\smallint \smallint 4\Pi \left[ {F_{1} \left( {\theta ,\varphi } \right) \cdot F_{2} \left( {\theta ,\varphi } \right)d\Omega } \right]} \right|^{2} }}{{\smallint \smallint 4\Pi \left| {F_{1} \left( {\theta ,\varphi } \right)} \right|^{2} d\Omega \smallint \smallint 4\Pi \left| {F_{2} \left( {\theta ,\varphi } \right)} \right|^{2} d\Omega }}$$

**Figure 20:**
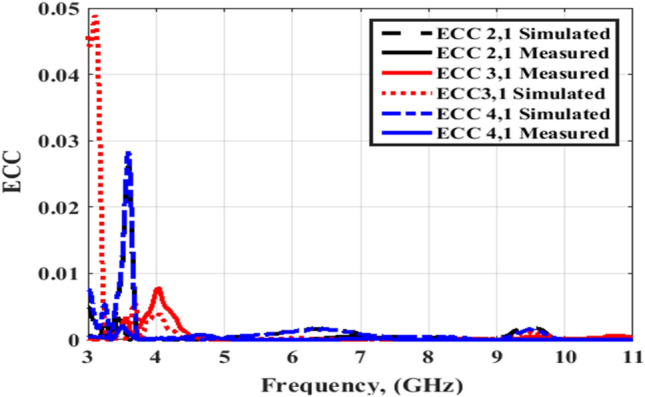
Port #1 ECC simulated and testing outcomes of the flexible MIMO antenna in the flat configuration.

**Figure 21:**
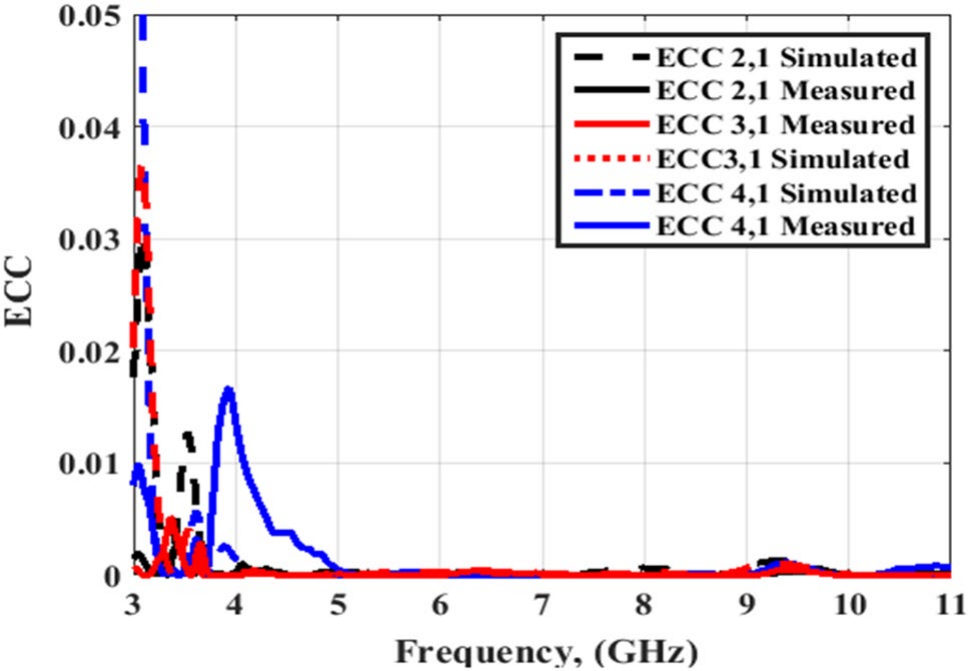
Port #1 ECC simulated and testing outcomes of the flexible MIMO antenna in bending configuration with R = 45 mm.

**Figure 22:**
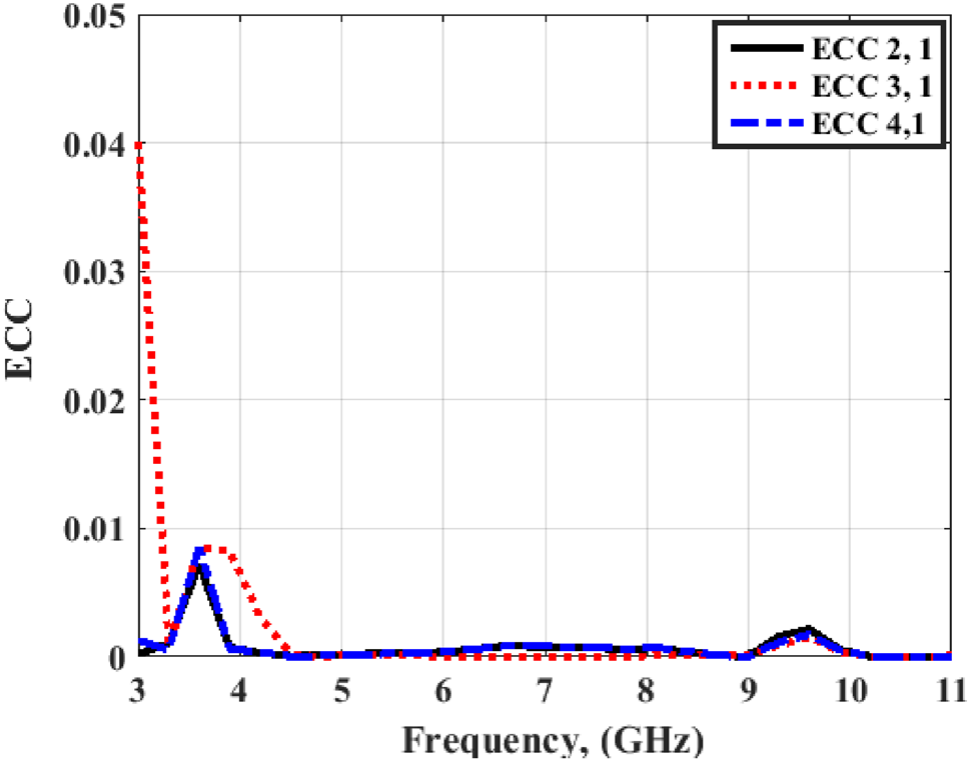
Port #1 ECC outcomes of the flexible MIMO antenna in the flat configuration.

**Figure 23:**
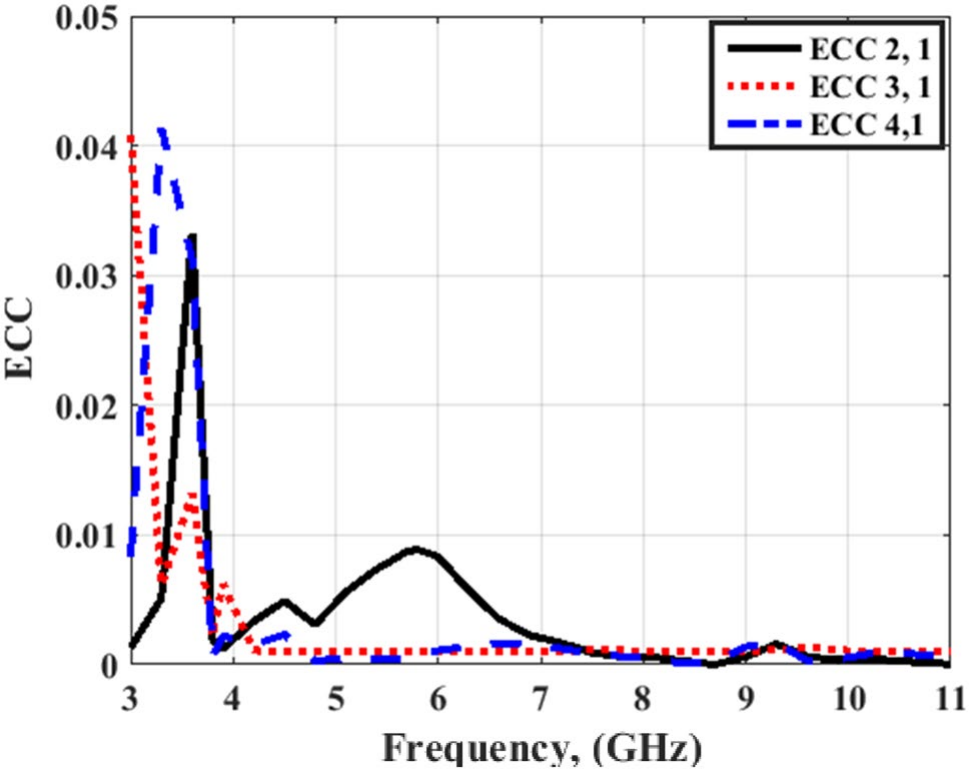
Port #1 ECC outcomes of the flexible MIMO antenna in bending configuration with R = 45 mm.

Figures 20 and 21 illustrate the ECC simulated and tested outcomes of the flexible MIMO antenna for both flat and bending configurations. The value of the ECC is lower than 0.03 from 3.5 to 4 GHz and lower than 0.01 from 4 to 11 GHz. As well, Figs. 22 and 23 illustrate the ECC the outcomes of the flexible MIMO antenna for both flat and bending configurations extracted from the far field's results. The value of the ECC is lower than 0.04 from 3.5 to 4 GHz and lower than 0.01 from 4 to 11 GHz.

The DG is linked with the ECC over Eq. (3) to confirm the MIMO system diversity^26^.

3 $${\text{DG}} = 10{*}\sqrt {1 - \left| {{\text{ECC}}} \right|}$$

**Figure 24:**
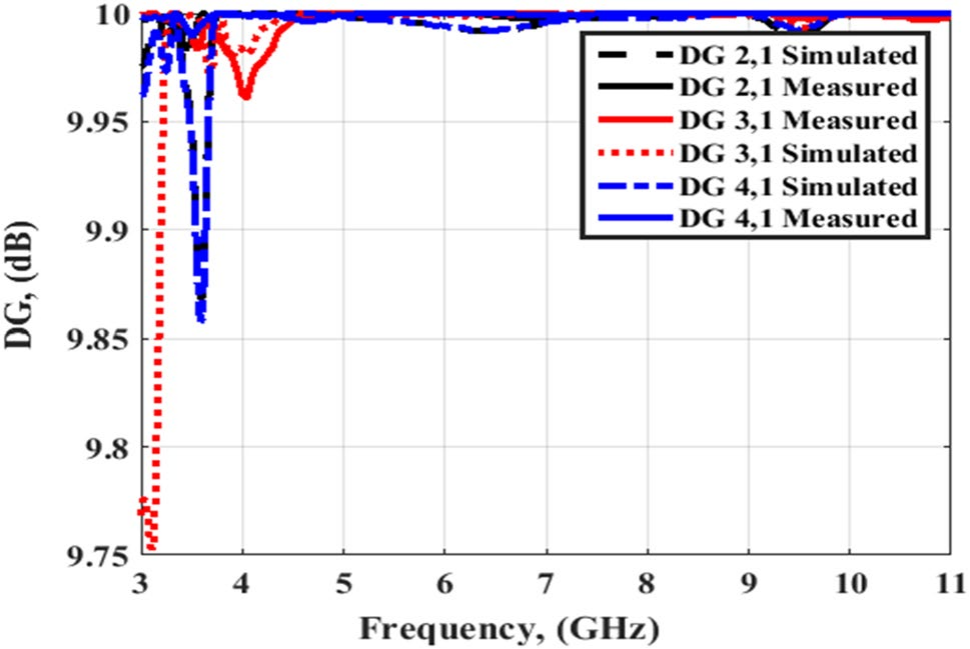
Port #1 DG simulated and testing outcomes of the flexible MIMO antenna in the flat configuration.

**Figure 25:**
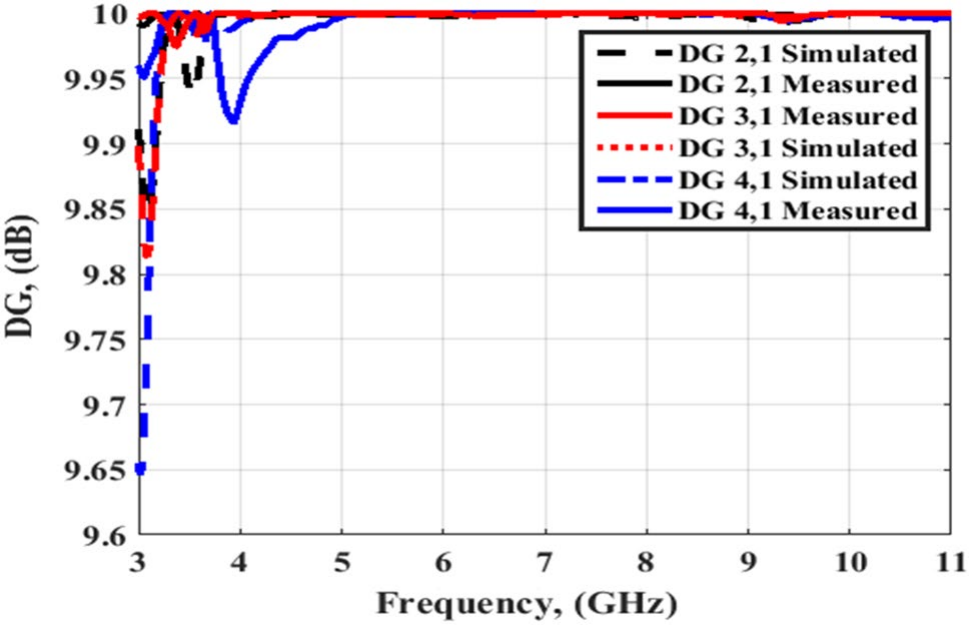
Port #1 DG simulated and testing outcomes of the flexible MIMO antenna in bending configuration with R = 45 mm.

The simulated and tested DG outcomes for both flat and bending configurations are displayed in Figs. 24 and 25. The DG outcomes have values ≥ 9.9 dB through the designed working band. The CCL (Bit/S/Hz) is the last MIMO parameter and its value can be calculated by utilizing Eqs. (4), (5)^26^

4 $${\text{C}}\left( {{\text{Loss}}} \right) = - \log_{2} \det \left( {{\uppsi }^{{\text{R}}} } \right)$$

5 $$\begin{gathered} {\uppsi }^{{\text{R}}} = \left[ {\begin{array}{*{20}c} {{\uprho }11} & {{\uprho }12} \\ {{\uprho }21} & {{\uprho }22} \\ \end{array} } \right],{\uprho }_{{{\text{ii}}}} = 1 - \left( {\left| {{\text{S}}_{{{\text{ii}}}} } \right|^{2} + \left| {{\text{S}}_{{{\text{ij}}}} } \right|^{2} } \right) \hfill \\ \;{\text{and}}\; \hfill \\ {\uprho }_{{{\text{ij}}}} = - \left( {{\text{S}}_{{{\text{ii}}}}^{*} {\text{S}}_{{{\text{ij}}}} + {\text{S}}_{{{\text{ji}}}}^{*} {\text{S}}_{{{\text{ij}}}} } \right),\;{\text{for}}\;{\text{i}},\;{\text{j}} = 1\;{\text{or}}\;2 \hfill \\ \end{gathered}$$

**Figure 26:**
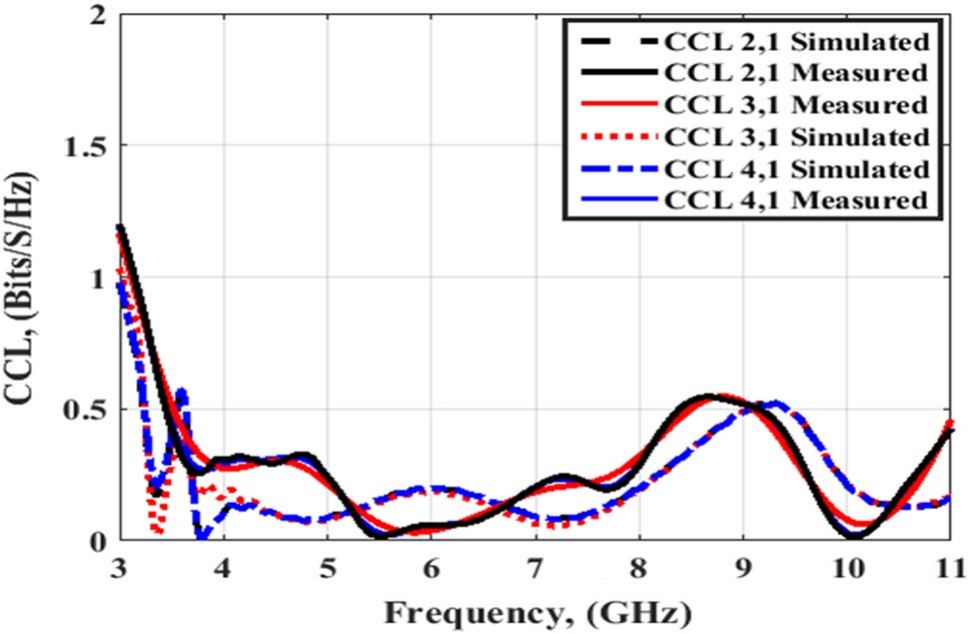
Port #1 CCL simulated and testing outcomes of the flexible MIMO antenna in the flat configuration.

**Figure 27:**
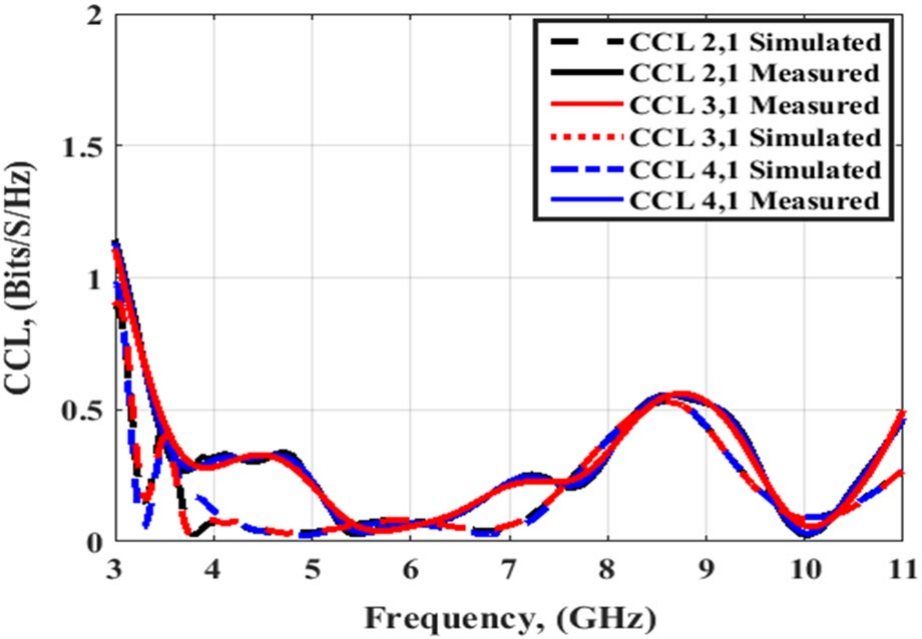
Port #1 CCL simulated and testing outcomes of the flexible MIMO antenna in bending configuration with R = 45 mm.

Figures 26 and 27 display the simulated and tested CCL outcomes for both flat and bending configurations. The CCL fulfills a value ≤ 0.5 bit/s/Hz over the worked band. From the outcomes presented in Figs. 20, 21, 22, 23, 24, 25, 26 and 27, it is seen good trend between the simulated and tested outcomes can be realized with a slight shift due to the previously tested S-parameters results which are used in the calculations. A flexible MIMO antenna in comparison with other flexible and rigid designs to confirm the performance of the suggested MIMO antenna is tabulated in Table 2. It is observed that the suggested flexible MIMO antenna has a miniaturized size with a suitable level of isolation, gain, and ECC values which support it in the UWB flexible networks applications.

**Table 2:** The flexible MIMO antenna in comparison with other flexible and rigid designs.

References	Size (mm^2^)/λo × λo	εr/thickness (mm)	No of ports	BW (GHz)	Gain (dBi)	Isolation (dB)	ECC/ DG	Flexibility	Technique method
^13^	42 × 42 0.44 × 0.44 @ 3.2 GHz)	4.4/1.6	4	3.2–12	4	≥ 17	0.01/9.96	No	Stub
^14^	81 × 87 0.81 × 0.87 @ 3.03 GHz)	4.4/1.6	4	3.03–10.74	5	≥ 20	0.1/–	No	Orthogonal
^15^	40 × 40 0.38 × 0.38 @ 2.9 GHz)	4.4/1.57	4	2.9–14	4	17	0.03/–	No	Orthogonal
^16^	36 × 36 0.37 × 0.37 @ 3.1 GHz)	4.4/1	4	3.1–10.6	3.5	15	0.02/–	No	Stub
^17^	25 × 25 0.3 × 0.3 @ 3.7 GHz)	4.4/1.6	2	3.7 -9	3.8	15	< 0.01/9.8	No	DGS
^18^	50 × 30 0.41 × 0.25 @ 2.5 GHz)	4.4/1.6	2	2.5–14.5	4	> 20	< 0.04/8	No	Stub
^19^	110 × 97 0.55 × 0.48 @ 1.5 GHz)	Jeans 1.7/1.4	4	1.5—3.8/ 4.1 to 6.1	4	25–33	< 0.1/9.98	Yes	Stub
^20^	16 × 71.5 0.17 × 0.76 @ 3.2 GHz)	2.2/0.254	4	3.2—14	5	> 22	< 0.006/9.88	Yes	Sideby Side
^21^	38.1 × 38.1 0.3 × 0.3 @ 2.4 GHz)	Felt 1.2/2	2	2.4–2.485	2.7	≥ 12 dB	< 0.01/–	Yes	Orthogonal
^22^	22 × 31 0.21 × 0.29 @ 2.9 GHz)	Kapton 3.4/0.125	2	2.9–12	2.6	> 15	< 0.3/–	Yes	Stub
^23^	30 × 56.5 0.24 × 0.45 @ 2.4 GHz)	LCP 2.9/0.1	2	2.4–11.3	5.35	> 23	0.008/9.99	Yes	Stub
This work	54 × 54 0.63 × 0.63 @ 3.5 GHz)	3/0.13	4	3.5–11	4.5	≥ 17	< 0.03/9.98	Yes	Orthogonal

## Conclusion

A flexible single and quad-port MIMO CPW fed antennas with crescent-shaped have been successfully investigated. The single unit has been designed fabricated and tested as well; the quad-port has been added orthogonally and tested using VNA. The radiation characteristics and MIMO parameters in form of peak gain, radiation patterns DG, ECC, and CCL have been discussed to validate the suggested antenna. The flexible MIMO antenna has been achieved S_11_ < − 10 dB from 3.5 GHz up to 11 GHz with mutual coupling ≤ − 17 dB between ports. As well, the MIMO antenna performance under bending situations has been discussed to ensure the flexible behavior of the antenna response. Finally, a comparison between the suggested antenna with others has been employed which supports it to be utilized in the flexible UWB networks.

For future work, this work can be extended to work in smart wearable applications where it has a compact and flexible design. Also, many ports with suitable isolation can be utilized to enhance the channel capacity of the MIMO system.

## Data Availability

All data generated or analyzed during this study are included in this article (and there are no supplementary materials).
